# Screening of Microorganisms Producing Cold-Active Oxidoreductases to Be Applied in Enantioselective Alcohol Oxidation. An Antarctic Survey

**DOI:** 10.3390/md9050889

**Published:** 2011-05-24

**Authors:** Lidiane S. Araújo, Edna Kagohara, Thaís P. Garcia, Vivian H. Pellizari, Leandro H. Andrade

**Affiliations:** 1 Instituto de Química, Universidade de São Paulo, Av. Prof. Lineu Prestes 748, SP 05508-900, São Paulo, Brazil; E-Mails: lidianepiaui@yahoo.com.br (L.S.A); ednakb@iq.usp.br (E.K.); thais_pelegrin@yahoo.com.br (T.P.G); 2 Instituto Oceanográfico, Universidade de São Paulo, Praça do Oceanográfico 191, SP 05508-120, São Paulo, Brazil; E-Mail: vivianp@usp.br (V.H.P.)

**Keywords:** oxidoreductases, chiral alcohols, psychrophile, psychrotroph, oxidation, deracemisation

## Abstract

Several microorganisms were isolated from soil/sediment samples of Antarctic Peninsula. The enrichment technique using (*RS*)-1-(phenyl)ethanol as a carbon source allowed us to isolate 232 psychrophile/psychrotroph microorganisms. We also evaluated the enzyme activity (oxidoreductases) for enantioselective oxidation reactions, by using derivatives of (*RS*)-1-(phenyl)ethanol as substrates. Among the studied microorganisms, 15 psychrophile/psychrotroph strains contain oxidoreductases that catalyze the (*S*)-enantiomer oxidation from racemic alcohols to their corresponding ketones. Among the identified microorganisms, *Flavobacterium* sp. and *Arthrobacter* sp. showed excellent enzymatic activity. These new bacteria strains were selected for optimization study, in which the (*RS*)-1-(4-methyl-phenyl)ethanol oxidation was evaluated in several reaction conditions. From these studies, it was observed that *Flavobacterium* sp. has an excellent enzymatic activity at 10 °C and *Arthrobacter* sp. at 15 and 25 °C. We have also determined the growth curves of these bacteria, and both strains showed optimum growth at 25 °C, indicating that these bacteria are psychrotroph.

## 1. Introduction

Enantiomerically pure alcohols are important precursors in the synthesis of bioactive compounds, especially in agrochemical and pharmaceutical industries [1]. Biocatalytic transformation, an enzyme-catalyzed reaction, is one of the techniques that have been rapidly developed in the last years in the synthesis of chiral alcohols. The most employed enzymes for this process are alcohol dehydrogenases, a subclass of oxidoreductases [2,3]. The biocatalytic reactions can be performed by isolated enzymes or whole-cell system, which may be microorganisms or plant cells [4]. It is well known that the screening of a wide variety of microorganisms living in the soil is an efficient method to obtain the desired enzyme [5–7]. In this context, unusual sources, such as microorganisms from extreme environments, have called the scientists attention for the possibility to find new biocatalysts or natural products [8]. Psychrophile microorganisms contain cellular processes that are thoroughly adapted to survival and proliferation at low temperatures and rates similar to those achieved by the closely related species living in temperate environments. This implies that they have developed various structural and physiological adjustments, enabling them to compensate the deleterious effects of low temperatures. The two main physicochemical low temperatures effects are: chemical rates decrease exponentially with decreasing temperature; temperature has also a strong effect on the viscosity of the medium, thereby contributing to further slow down the reaction rates [9]. Several cold-adapted enzymes from Antarctic bacterial strains have been studied [10–14]. The specific activities of these enzymes are much higher than their mesophilic counterparts [9]. This is reflected by the significantly lower activation energy values and the significant enzyme flexibility improvement, leading to a large distribution of conformational isomers [15] accompanied by increased thermolability. In cold-adapted enzymes, flexibility appears to play a crucial role in catalysis by securing the accommodation of the substrate at low temperature, as well as by facilitating water molecules movement and products release [9].

In this work we isolated several microorganisms from soil and sediment samples collected in the Antarctic Continent to be applied in enantioselective oxidation of (*RS*)-1-phenylethanol derivatives. The main goal of this work was to select microorganisms producing cold-active oxidoreductases to perform alcohol oxidation in an enantioselective fashion.

## 2. Results and Discussion

### 2.1. Isolation of Microorganisms Producing Oxidoreductases

By using the enrichment technique 232 microorganisms from soils or sediments samples were isolated (Table 1). This technique consisted of applying (*RS*)-1-phenylethanol (1 mM) as a carbon source presented in the culture medium used in the isolation of microorganisms [16]. The use of this chiral alcohol results in the isolation of microorganisms containing new oxidoreductases that can be applied in enantioselective alcohol oxidation. The enzymatic system able to perform the alcohol oxidation can be consisting of a dehydrogenase or an oxidase. However, after the enzyme purification, we will be able to elucidate the enzyme responsible for the alcohol oxidation reaction.

The results described in Table 1 demonstrated that the highest number of microorganisms was obtained from soil samples and culture medium at neutral pH. The culture medium MCFE7 chemical composition was aimed to induce the isolation of fungi. The minimal medium culture MCBE7 was chosen to induce the isolation of yeasts and bacteria. Despite the pH difference between the culture media, it was possible to observe bacteria and fungi growth in both media.

### 2.2. Evaluation of the Enzyme Activity of Microorganisms

**Scheme 1.**
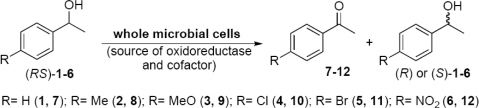
Enantioselective oxidation of (*RS*)-1-phenylethanol and its derivatives.

Enantioselective oxidations of alcohols, *para*-substituted (*RS*)-1-phenylethanols, were used to evaluate the enzyme activity (oxidoreductases, OXRED) of 232 microorganisms psychrophile/psychrotroph previously isolated (Scheme 1).

From this study, 15 microorganisms presented oxidoreductases that catalyzed the oxidation of the (*S*)-enantiomer of racemic alcohols to their corresponding ketones. Therefore, the (*R*)-alcohols remained intact with high enantiomeric excesses (up to >99%). It was considered as good enzyme activity (OXRED), conversions of alcohol to ketone > 10% and enantiomeric excess >60%. Some of the results are shown in Table 2.

As shown in Table 2, the enzyme activities of the microorganisms were very high when the R substituent in alcohols (**1**–**6**) was a methoxy group. In these cases, considering a kinetic resolution process, the alcohols conversions to their corresponding ketones were excellent (Entries 3, 9, 21 and 33), and in some cases the enantiomeric excesses were higher than 99% (Entries 3, 9 and 21). It is noteworthy that, in a reaction of kinetic resolution the maximum conversion is 50%.

In reactions mediated by bacterium HK1D10 cells, in which the R substituent was halogen, low conversions (∼1%) and no selectivity were observed (Entries 34 and 35). In general, all microorganisms produce oxidoreductases with preferential oxidation for the (*S*)-enantiomer from the (*RS*)-alcohols (**1**–**6**)

Based on these results we decided to perform only the identification of microorganisms that showed excellent enzyme activity. For this purpose *Flavobacterium* sp. (HK1D9) and *Arthrobacter* sp. (MPS8D3) were selected. These bacteria were isolated from soil and sediment samples, respectively.

**Figure 1.**
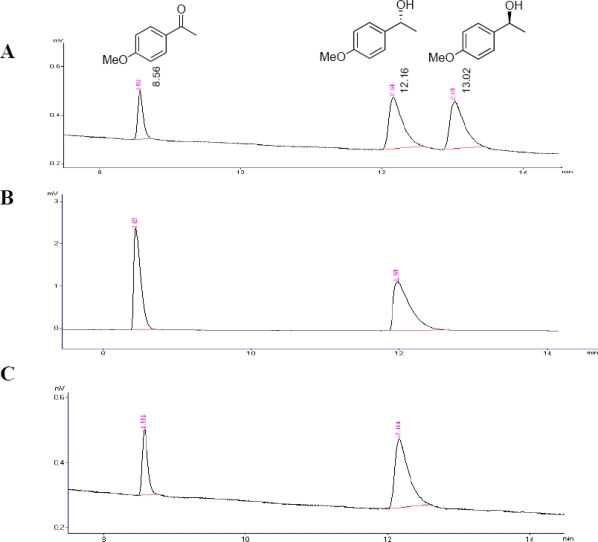
Analysis using GC. **(a)** Sample containing a mixture of (*RS*)-1-(4-methoxyphenyl)ethanol and 4-methoxyacetophenone. **(b)** Sample from oxidation reaction of (*RS*)-**3** mediated by *Arthrobacter* sp. **(c)** Sample from oxidation reaction of (*RS*)-**3** mediated by *Flavobacterium* sp.

The chromatograms from oxidation reaction of the compound (*RS*)-**3** mediated by cells of *Flavobacterium* sp. and *Arthrobacter* sp. (Table 2, Entries 3 and 9) are shown in Figure 1, as an example of the chiral analysis using GC employed for this enzyme evaluation step.

According to the chromatogram obtained from oxidation reaction of the (*RS*)-**3** mediated by *Arthrobacter* sp. (Figure **1b**) and *Flavobacterium* sp. (Figure **1c**), it can be seen that the (*S*)-enantiomer was oxidized, leaving the unreacted alcohol (*R*)-**3** with high enantiomeric excess.

### 2.3. Optimization of the Enantioselective Oxidation Reaction Using *Flavobacterium* sp. and *Arthrobacter* sp

*Flavobacterium* sp. and *Arthrobacter* sp. were selected to be applied in the optimization study due to their high enantioselective performance in the oxidation reaction of the alcohols (*RS*)-**1**–**6**. Based on the good results presented in Table 2 (Entries 2, 8, 14, 20) and by the fast GC analysis using a chiral column, we selected (*RS*)-1-(4-methylphenyl)ethanol as model substrate for this optimization study.

A survey in the literature indicated that psychrophile/psychrotroph microorganisms tolerate temperatures between 0 and 40 °C [17–22]. The temperature corresponds to one of the major environmental factors that can influence the bacterial growth, when the temperature is increased, the chemical and enzymatic reactions in cells tend to become faster, thus accelerating the growth rate [23].

In order to determine the ideal reaction conditions for achieving an excellent kinetic resolution process mediated by *Flavobacterium* sp. (HK1D9) and *Arthrobacter* sp. (MPS8D3), the bacterial cells were resuspended in phosphate buffer solution (Na_2_HPO_4_/KH_2_PO_4_, pH 7.0, 0.1 mol/L).

The selected temperatures were 5, 10, 15, 20, 25 and 30 °C (each one is tolerated by psychrotroph microorganisms) [18]. These temperatures were used for both bacterial growth and alcohol oxidation reaction. The results are shown in Table 3.

**Scheme 2.**
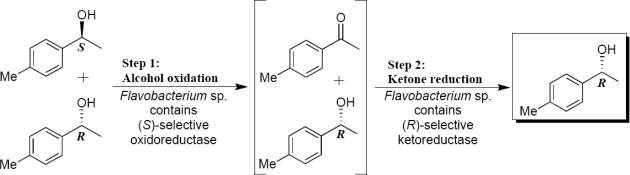
Deracemisation process mediated by *Flavobacterium* sp. (HK1D9).

When the bacterial growing and reactions were performed at 15 °C and 25 °C (*Flavobacterium* sp.) excellent oxidoreductase activities were observed. The best conversion of the (*RS*)-alcohol **2** to ketone (45%) was obtained after 24 h reaction at 25 °C (Table 3, Entry 13,). Along the reaction, there was a few decrease in ketone concentration and an increase in (*R*)-1-(4-methylphenyl)ethanol concentration and, consequently its enantiomeric excess (Table 3, Entries 13–15). In an oxidative kinetic resolution process, the ratio of the ketone and a single alcohol enantiomer concentration should be 1:1. The above mentioned behavior indicates the presence of ketoreductase that catalyzes enantioselectively the reduction of the produced 4-methylacetophenone to the (*R*)-1-(4-methylphenyl)ethanol, resulting in a higher (*R*)-alcohol concentratrion than 50%. This process can be called as deracemisation (Scheme 2).

The *Arthrobacter* sp. (MPS8D3) showed good enzyme activity at 15, 20 and 25 °C (24 h), since under these temperatures, the highest enantiomeric excess and concentration of the (*R*)-1-(4-methylphenyl)ethanol were obtained (Table 3, Entries 25, 28 and 31). On the other hand, it was observed that *Flavobacterium* sp. and *Arthrobacter* sp. exhibited very low enzyme activity at 10 °C, since the values of ketone conversions and alcohol e.e. were very low (Table 3, Entries 4–6 and 22–24).

It is noteworthy to mention that there is few deracemisation process described in the literature performed by a single microorganism [3,24–28] or without the aid of chemical agents [29,30]. This phenomenon is extremely interesting to be pursued in bioprospecting process.

We decided to study the oxidation of (*RS*)-1-(4-methylphenyl)ethanol at different temperatures (5, 10, 15, 20, 25, 30 °C) after growthing the *Flavobacterium* sp. and *Arthrobacter* sp. at a specific temperature, 15 or 25 °C. The bacterial growth temperatures were selected due to the good results of conversions and enantioselectivities of the alcohol oxidation (Table 3, Entries 7–9, 13–17 and 31–33). The main goal of this study was to evaluate the enantioselectivity of oxidoreductases produced by bacterial growth at 15 and 25 °C in the oxidation reaction carried out at different temperatures (Tables 4 and 5).

Analyzing the data in Table 4, in which *Flavobacterium* sp. grew at 25 °C, we found low enzyme activity for the oxidation of (*R*,*S*)-1-(4-methylphenyl) ethanol at 30 °C leading to low ketone conversion and enantioselectivity (Entries 16–18). However, this bacterium showed good enzyme activity in other temperatures (Entries 1–15). In general there was an increase in conversion and enantioselectivity with increasing reaction time. However, the best oxidation performance was observed at 10 °C and 72 h of reaction (Table 4, Entry 6). In this case, a perfect kinetic resolution process was achievied, ketone conversion 50% and alcohol e.e. > 99%.

The reactions carried out under several temperatures and mediated by cells of *Arthrobacter* sp. resulted in moderate to excellent alcohol e.e. and ketone conversion (Table 4, Entries 19–36). For example, oxidation reactions carried out at 15 to 30 °C, there was a decrease in ketone concentration and an increase in alcohol e.e. over the reaction time (Table 4, Entries 25–36). The *Flavobacterium* sp. has oxidoreductase with high activity at 10 °C and *Arthrobacter* sp. at 15 and 20 °C.

When the microbial growing temperature was 15 °C (*Flavobacterium* sp.) a low enzyme activity was observed at 5 and 30 °C. These reactions afforded low values of ketone concentration and enantioselectivies (Table 5, Entries 1–3 and 16–18). However, the best ketone concentration and enantioselectivity were observed for reactions carried out at 15 and 20 °C (Table 5, Entries 9 and 12).

On the other hand, *Arthrobacter* sp. showed good enzyme activity at 5 and 10 °C after 48 and 72 hours, since it was obtained high enantiomeric excess for the alcohol (*R*)-**2**, up to >99% (Table 5, Entries 20, 21, 23 and 24). We also can see that *Arthrobacter* sp. showed good enzymatic activity at reaction temperatures of 15–30 °C, in which ketone conversions and e.e. for the alcohol (*R*)-**2** were moderate to excellent (Table 5, Entries 25–36). At these temperatures, the value of (*R*)-alcohol concentration is higher than 50% with increasing the reaction time. As observed for *Flavobacterium* sp. (Table 3 and Scheme 2), this process indicates the presence of ketoreductase that catalyzes enantioselectively the reduction of the produced 4-methylacetophenone to the (*R*)-1-(4-methylphenyl)ethanol, resulting a high (*R*)-alcohol concentration.

In general, it was observed that *Arthrobacter* sp. and *Flavobacterium* sp. had excellent enzyme activity with growth temperature at 25 °C.

**Figure 2.**
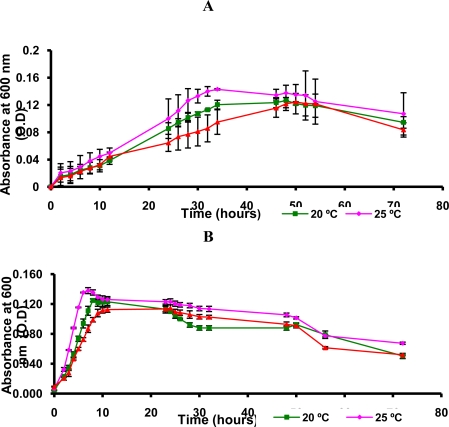
Spectrophotometric analysis of optical density (OD) of the culture of *Flavobacterium* sp. **(A)** and *Arthrobacter* sp. **(B)**. Culture medium (600 mL), (*RS*)-1-(phenyl)ethanol (1 mmol/L), 160 rpm.

Therefore, we decided to determine the growth curves of *Flavobacterium* sp. and *Arthrobacter* sp. at 15, 20 and 25 °C (Figure 2). By spectrophotometric analysis of optical density (OD) of bacterial culture it was evaluated the turbidity and estimated the relative concentration cell.

According to the Figure 2, it was noted that *Flavobacterium* sp. and *Arthrobacter* sp. grew well at 25 °C and exhibited excellent enzyme activity at this temperature (Table 4, Entries 13–15 and 31–33). Then, it was concluded that these bacteria are psychrotroph since it excludes the possibility of psychrophile, in which the maximum temperature for growth is 20 °C. This conclusion was based on literature data about the identification of psychrotroph microorganism [17,31,32].

## 3. Experimental Section

### 3.1. General Methods

Acetophenone, 1-(4-methylphenyl)ethanone, 1-(4-methoxyphenyl)ethanone, 1-(4-bromophenyl) ethanone, 1-(4-chlorophenyl)ethanone and 1-(4-nitrophenyl)ethanone were purchased from Aldrich and used without further purification. Thin-layer chromatography (TLC) was performed using pre-coated plates (Aluminum foil, silica gel 60 F_254_ Merck, 0.25 mm). Silica gel (Acros Organics, 0.035–0.070 mm, 240–400 mesh) was used for flash chromatography. Low Resolution Mass Spectra (LRMS) were recorded on a Shimadzu GCMS P5050A (70 eV) spectrometer and GC cappillary column used was a DB-5-MS (30 m × 0.25 mm × 0.25 μm, J&W Scientific), carrier gas-He, 100 kPa): injector 270 °C; detector 270 °C. Temperature program 100–250 °C, rate 10 °C/min. GC analyses were performed in a Shimadzu GC-17A instrument with a FID detector using hydrogen as carrier gas (100 kPa), autosampler AOC20i. It was used a GC chiral cappillary column, Chirasil-Dex CB β-cyclodextrin (25 m × 0.25 mm, Varian), for determination of the conversion and enantiomeric excesses. Sterile materials were used to perform the experiments and the microorganisms were handled in a laminar flow cabinet Fisher Hamilton *Class II Biological Safety Cabinet*.

### 3.2. Preparation of the (RS)-1-(Phenyl)ethanols (**1**–**6**) [33]

To a solution of acetophenones (**7**–**12**) (10 mmol) in methanol (20 mL) was added NaBH_4_ (11.1 mmol, 420 mg), and the mixture was stirred at room temperature for 4 h. The methanol was removed by vacuum evaporation. Distilled water (20 mL) was added into the reaction mixture and the pH adjusted to 6 with aqueous solution of HCl (1 mmol/L), then the aqueous layer was extracted with methylene dichloride (3 × 30 mL). The organic phases were combined and dried over MgSO_4_. The solvent was removed in vacuum and the residue was purified by silica gel column chromatography eluting with a mixture of hexane and ethyl acetate (4:1) to afford compounds (*RS*)-**1**–**6**. The ^1^H NMR and *^13^*C NMR spectra of these compounds were in agreement with those reported in the literature [34,35].

### 3.3. Procedures for Bioprospection

#### 3.3.1. Soil and Sediment Sampling Procedure

Soil and sediment samples were collected from environmental preservation area in Antarctic Peninsula (Admiralty Bay, King George Island, Antarctic in 2008): Peninsula Keller (21 E 427302, UTM 3115830); (21 E 425986, UTM 3117583); (21 E 426110, UTM 3118275); Machu Picchu (21 E 423394, UTM 3114505); Ponta Hennequin (21 E 427680, UTM 3111880); Arctowski (21 E 423807, UTM 3106862); Ponta Demay (21 E 425211, UTM 3100473); Ponta Botany (21 E 424432, UTM 3092107); Ponta Steinhouse (21 E 425419, UTM 3110733); The samples were collected under clean, sterile conditions and kept frozen in polyethylene tubes at −20 °C.

#### 3.3.2. Culture Media

The culture media were (amount of the ingredients are indicated per liter):

-MCFE7 (pH = 5.6) = (*RS*)-1-(phenyl)ethanol–1 mmol, 0.122 g, 122 μL), KH_2_PO_4_ (0.1%; 1.0 g), MgSO_4_.7H_2_O (0.04%; 0.4 g), NH_4_Cl (0.04%; 0.4 g), Dextrose (Oxoid) (0.025%; 0.25 g), Peptone (Oxoid) (0.025%; 0.25 g), agar (Oxoid) (2.0%; 20.0 g; when needed).
-MCBE7 (pH = 7.0)= (*RS*)-1-(phenyl)ethanol–1 mmol, 0.122 g, 122 μL), K_2_HPO_4_ (0.2%; 2.0 g), KH_2_PO_4_ (0.1%; 1.0 g), MgSO_4_.7H_2_O (0.04%; 0.4 g), NH_4_Cl (0.04%; 0.4 g), Meat Extract (Oxoid) (0.025%; 0.25 g), Peptone (Oxoid) (0.025%; 0.25 g), agar (Oxoid) (2.0%; 20.0 g; when needed).

#### 3.3.3. Isolation and Growth of Microorganisms

A soil suspension was prepared by adding 20 mL of sterile phosphate buffer solution (pH 7.0, 0.1 mol/L) to 2.0 g of the soil or sediments sample. It was stirred, the solution resting for about 1 minute for sedimentation and 200 μL of the supernatant were incubated (in duplicate) in a polypropylene microtiter plate (each well consisting of a 10 mL total volume capacity; Whatman) containing 2 mL of the appropriate culture media:

- One microtiter plate contains MCFE7 culture medium;
- One microtiter plate contains MCBE7 culture medium;

After 4 days of incubation for MCFE7-containing microtiter plate and 2 days of incubation for MCBE7-containing microtiter plate at 20 °C, aliquots of 100 μL of microorganisms culture from each well was sprayed onto the culture medium agar plates to microorganisms isolation step.

From each microorganism culture sample (100 μL) that was plated onto solid culture medium, individual colonies appeared. Each colony was inoculated separately into 20 mL of appropriate culture media in Erlenmeyer flasks (125 mL) and incubated in an orbital shaker during 2 or 4 days (160 rpm, 20 °C).

### 3.4. Enzyme Assays

From each pre-culture obtained previously, a 750 μL aliquot was transferred to microtubes containing 250 μL of glycerol for cryo-preservation (−20 °C). Another 200 μL were transferred to inoculate in a sterile polypropylene microtiter plate containing 2 mL of culture media per well. The plates were kept at 20 °C in an orbital shaker (160 rpm) during 2 or 4 days and then 40 μL of a solution of each alcohol (**1**–**6**, 5 mmol/L) in dimethylformamide were added to each well of the plate. The microtiter plates were returned to the same incubation condition as described above for 72 hour. Each well content was extracted by stirring with *tert-*butyl methyl ether (2.0 mL) followed by centrifugation (6000 rpm, 1 min.). The organic phase was analyzed by GC using a chiral capillary column (see Section 3.5).

### 3.5. GC Analysis

GC conditions (carrier gas-H_2_, 100 kPa): injector 220 °C; detector 220 °C. Isotherm and *t*_R_ (min): retention time of chiral compounds: (*RS)*-phenyl-ethanol: 107 °C, *R*-enantiomer 6.22 min, *S*-enantiomer 6.87 min; (*RS)*-1-(4-methyl-phenyl)-ethanol: 113 °C, *R*-enantiomer 6.79 min, *S*-enantiomer 7.71 min; (*RS)*-1-(4-methoxy-phenyl)-ethanol: 119 °C, *R*-enantiomer 6.22 min, *S*-enantiomer 6.87 min; (*RS)*-1-(4-chloro-phenyl)-ethanol: 130 °C, *R*-enantiomer 7.33 min, *S*-enantiomer 8.19 min; (*RS)*-1-(4-bromo-phenyl)-ethanol: 135 °C, *R*-enantiomer 9.54 min, *S*-enantiomer 10.52 min; (*RS)*-1-(4-nitro-phenyl)-ethanol: 150 °C, *R*-enantiomer 19.92 min, *S*-enantiomer 20.30 min. The enantiomeric excess (ee) was defined as the ratio of {([*R*] – [*S*])/([*R*] + [*S*])} × 100%, where [R] and [S] are the concentrations of (*R*) and (*S*) enantiomers, respectively.

### 3.6. Microorganisms Library and Preservation

The microorganism strains with the best performance in enzyme assays were stored in a freezer at −20 °C. They were preserved as suspensions in glycerol solution (20%) using two different culture media described in Section 3.3.2.

### 3.7. Bacteria Identification

The bacteria with the best performance in enzyme assays were identified using 16S rDNA sequencing analysis and comparison with GenBank top hits after a BLAST search [36].

### 3.8. Effect of Temperature and Reaction Time on the Oxidation Reactions Using *Flavobacterium* sp. and *Arthrobacter* sp

Cultures of *Flavobacterium* sp. and *Arthrobacter* sp. were separately inoculated under sterile conditions into 20 mL of appropriate culture media in Erlenmeyer flasks (125 mL) and incubated in an orbital shaker during 2 days (160 rpm, 20 °C). After that, the cultures were transferred separately to two Erlenmeyer flasks (2000 mL) containing 600 mL of sterilized culture media and incubated again. Then the cells were harvested by centrifugation at 20 °C on Sorvall RC-5B (Plus rotor) for 15 min at 5000 rpm. The harvested cells were resuspended in 60 mL of sterile phosphate buffer solution (Na_2_HPO_4_/KH_2_PO_4_, pH 7.0, 0.1 mol/L), divided equally in 6 Erlenmeyer flasks (125 mL) and an aliquot (200 μL) of substrate solution (*RS*)-1-(4-methylphenyl)ethanol (1 mmol/L) was added to each flask. The mixture was kept at different temperatures (5, 10, 15, 20, 25 and 30 °C) in an orbital shaker (160 rpm) during 1, 2 or 3 days. The products of the total reaction volume (10 mL) were extracted by stirring with *tert-*butyl methyl ether (2.0 mL) followed by centrifugation (6000 rpm, 1 min.). The organic phase was analyzed by GC using a chiral capillary column (see Section 3.5).

### 3.9. Growth Measurements

Growth curve experiments were performed with Erlenmeyer flasks (2000 mL) containing 1000 mL of sterilized culture media and 100 mL of inoculated cultures. The mixture was incubated in an orbital shaker (160 rpm, 15, 20 and 25 °C). Samples of culture (1 mL) were periodically removed at 120 min intervals and the growth was estimated spectrophotometrically (and expressed as optical density [O.D.]) at 600 nm with a Biospectro model sp-220 spectrophotometer using cells with a 1 cm light path.

The replicate values were averaged and the O.D. was plotted against time on linear coordinates.

#### 3.10. Absolute Configuration

The absolute configurations of all compounds were determined by comparison of the sign of the measured specific rotation with those in the literature [37–39].

## 4. Conclusions

In summary, the enrichment technique using (*RS*)-1-(phenyl)ethanol, as a carbon source, was a very important tool for the selection of microorganisms producing cold-active oxidoreductases. Samples of soil and sediment from the Antarctic provided 232 microorganisms. The enzyme activity (oxidoreductase) of these microorganisms was evaluated in the oxidation of racemic alcohols. Fifteen microorganisms presented oxidoreductases which catalyzed the oxidation of the (*S*)-enantiomer from racemic alcohol to its corresponding ketone. Therefore, the (*R*)-alcohol remained intact and with high enantiomeric excess (up to >99%). After the optimization study, *Flavobacterium* sp. showed excellent enzyme activity with temperature of growth and oxidation reaction at 25 °C and 10 °C, respectively. However, *Arthrobacter* sp. presented excellent enzyme activity with temperature of growth and oxidation reaction at 25 °C. After the determination of growth curves for *Flavobacterium* sp. and *Arthrobacter* sp., it was possible to classify them as psychrotroph, since the best temperature for bacterial growth was 25 °C and 20 °C, respectively. Further studies regarding enzyme purification are in progress in our laboratory.

## Figures and Tables

**Table 1.** Microorganisms isolated from soil and sediment samples.

**Culture medium**	**Microorganisms (soil)**	**Microorganisms (sediment)**
MCBE7^a^	103	17
MCFE7^b^	96	16

General conditions: 2 g soil or sediment;

a pH 7, 2 days of incubation at 20 °C;

b pH 5.6, 4 days of incubation at 20 °C.

**Table 2.** Evaluation of the enzymatic activity of microorganisms isolated from soil/sediment samples collected in the Antarctic. 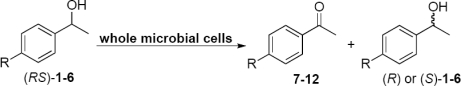

**Entry**	**Microorganism**	**R**	**Ketone/Alcohol^a^**	**Alcohol e.e. (%)^a^**
1	MPS8D3	H	11/89	25 (*R*)
2		Me	22/78	92 (*R*)
3		MeO	46/54	>99 (*R*)
4		Cl	21/79	89 (*R*)
5		Br	23/77	96 (*R*)
6		NO_2_	43/57	57 (*R*)

7	HK1D9	H	16/84	22 (*R*)
8		Me	27/73	97 (*R*)
9		MeO	47/53	>99 (*R*)
10		Cl	25/75	95 (*R*)
11		Br	4/96	17 (*R*)
12		NO_2_	34/66	33 (*R*)

13	HK2D2	H	6/94	10 (*R*)
14		Me	17/83	57 (*R*)
15		MeO	25/75	45 (*R*)
16		Cl	16/84	52 (*R*)
17		Br	21/79	61 (*R*)
18		NO_2_	7/93	6 (*R*)

19	MPS4D2	H	8/92	22 (*R*)
20		Me	17/83	93 (*R*)
21		MeO	44/56	>99 (*R*)
22		Cl	15/85	69 (*R*)
23		Br	19/81	87 (*R*)
24		NO_2_	21/79	22 (*R*)

25	HK1D8	H	2/98	3
26		Me	5/95	17 (*R*)
27		MeO	15/85	22 (*R*)
28		Cl	5/95	15 (*R*)
29		Br	5/95	15 (*R*)
30		NO_2_	5/95	15 (*R*)

31	HK1D10	H	11/89	17 (*R*)
32		Me	9/91	24 (*R*)
33		MeO	41/59	79 (*R*)
34		Cl	1/99	1
35		Br	1/99	2
36		NO_2_	4/96	2

Reaction conditions: (*RS*)-alcohols **1–6** (0.75 mmol), culture medium MCBE7 (pH 7.0, 2 mL), 20 °C, 160 rpm, 72 h.

a Determined by GC using a chiral column Chiral-Dex-CB; e.e.: enantiomeric excess.

**Table 3.** Oxidation of the (*RS*)-1-(4-methylphenyl)ethanol (**2**) mediated by *Flavobacterium* sp. and *Arthrobacter* sp. at different temperatures for bacterial growth and oxidation reaction.

			***Flavobacterium*sp. (HK1D9)**	

**Entry**	**Temp. (°C)^a^**	**Time (h)**	**Ketone/Alcohol^b^**	**Alcohol e.e. (%)^b^**
1	5	24	nd	nd
2		48	nd	nd
3		72	nd	nd

4	10	24	5/95	10 (*R)*
5		48	14/86	21 (*R)*
6		72	14/86	24 (*R)*

7	15	24	32/68	42 (*R)*
8		48	46/54	78 (*R)*
9		72	48/52	91 (*R)*

10	20	24	42/58	76 (*R)*
11		48	41/59	83 (*R)*
12		72	49/51	96 (*R)*

13	25	24	45/55	89 (*R)*
14		48	40/60	93 (*R)*
15		72	33/67	95 (*R)*

16	30	24	nd	nd
17		48	nd	nd
18		72	nd	nd

			***Arthrobacter*sp. (MPS8D3)**	

19	5	24	-	-
20		48	-	-
21		72	1/99	-

22	10	24	1/99	1
23		48	2/98	1
24		72	5/95	11 (*R)*

25	15	24	40/60	94 (*R)*
26		48	37/63	96 (*R)*
27		72	34/66	96 (*R)*

28	20	24	40/60	95 (*R)*
29		48	36/64	95 (*R)*
30		72	30/70	96 (*R)*

31	25	24	38/62	96 (*R)*
32		48	34/66	95 (*R)*
33		72	30/70	95 (*R)*

34	30	24	-	-
35		48	1/99	-
36		72	2/98	4

Reaction conditions: (*RS*)-alcohol **2** (0.75 mmol), cells harvested from culture medium (100 mL) and ressuspended in phosphate buffer solution (0.1 mol/L, pH 7.0, 10 mL), 5–30 °C, 160 rpm, 24–72 h;

a Temperature for bacterial growth and oxidation reaction;

b Determined by GC using a chiral column Chiral-Dex-CB; nd = not carried out due to very low biomass concentration.

**Table 4.** Growth of *Flavobacterium* sp. and *Arthrobacter* sp. at 25 °C and oxidation of (*RS*)-1-(4-methylphenyl)ethanol (**2**) at different reaction temperature.

			***Flavobacterium*sp. (HK1D9)**	

**Entry**	**Temp. (°C)^a^**	**Time (h)**	**Ketone/Alcohol^b^**	**Alcohol e.e. (%)^b^**
1		24	39/61	39 (*R*)
2	5	48	39/61	79 (*R*)
3		72	45/55	95 (*R*)

4		24	35/65	62 (*R*)
5	10	48	44/56	95 (*R*)
6		72	51/49	>99 (*R*)

7		24	25/75	36 (*R*)
8	15	48	43/57	83 (*R*)
9		72	43/57	95 (*R*)

10		24	28/72	49 (*R*)
11	20	48	35/65	80 (*R*)
12		72	38/62	83 (*R*)

13		24	45/55	89 (*R*)
14	25	48	40/60	93 (*R*)
15		72	33/67	95 (*R*)

16		24	7/93	6 (*R*)
17	30	48	6/94	9 (*R*)
18		72	7/93	18 (*R*)

			***Arthrobacter*sp. (MPS8D3)**	

19		24	40/60	88 (*R*)
20	5	48	44/56	96 (*R*)
21		72	43/57	96 (*R*)

22		24	40/60	94 (*R*)
23	10	48	46/54	97 (*R*)
24		72	41/59	96 (*R*)

25		24	41/59	>99 (*R*)
26	15	48	37/63	>99 (*R*)
27		72	36/64	>99 (*R*)

28		24	43/57	98 (*R*)
29	20	48	38/63	98 (*R*)
30		72	36/64	>99 (*R*)

31		24	45/55	89 (*R*)
32	25	48	40/60	93 (*R*)
33		72	33/67	95 (*R*)

34		24	42/58	97 (*R*)
35	30	48	33/67	97 (*R*)
36		72	27/73	97 (*R*)

Reaction conditions: (*RS*)-alcohol **2** (0.75 mmol), cells harvested from culture medium (100 mL) and ressuspended in phosphate buffer solution (0.1 mol/L, pH 7.0, 10 mL), 5–30 °C, 160 rpm, 24–72 h; Temperature for bacterial growth = 25 °C;

a Temperature for oxidation reaction;

b Determined by GC using a chiral column Chiral-Dex-CB.

**Table 5.** Growth of the *Flavobacterium* sp. and *Arthrobacter* sp. at 15 °C and oxidation reaction of the (*RS*)-1-(4-methylphenyl)ethanol (**2**) at different reaction temperatures.

		***Flavobacterium*sp. (HK1D9)**		

**Entry**	**Temp. (°C)^a^**	**Time (h)**	**Ketone/Alcohol^b^**	**Alcohol e.e. (%)^b^**
1	5	24	5/95	15 (*R*)
2		48	16/84	30 (*R*)
3		72	21/79	43 (*R*)

4	10	24	19/81	21 (*R*)
5		48	34/66	65 (*R*)
6		72	42/58	89 (*R*)

7	15	24	25/75	36 (*R*)
8		48	43/57	83 (*R*)
9		72	43/57	95 (*R*)

10	20	24	16/84	44 (*R*)
11		48	34/66	73 (*R*)
12		72	41/59	96 (*R*)

13	25	24	27/73	53 (*R*)
14		48	33/67	77 (*R*)
15		72	34/66	87 (*R*)

16	30	24	9/91	9 (*R*)
17		48	9/91	10 (*R*)
18		72	6/94	12 (*R*)

			***Arthrobacter*sp. (MPS8D3)**	

19	5	24	42/58	90 (*R*)
20		48	42/58	>99 (*R*)
21		72	45/55	>99 (*R*)

22	10	24	41/59	95 (*R*)
23		48	41/59	97 (*R*)
24		72	43/47	97 (*R*)

25	15	24	35/65	89 (*R*)
26		48	34/66	97 (*R*)
27		72	31/69	98 (*R*)

28	20	24	42/58	98 (*R*)
29		48	36/64	98 (*R*)
30		72	33/67	98 (*R*)

31	25	24	43/57	>99 (*R*)
32		48	36/64	>99 (*R*)
33		72	32/68	>99 (*R*)

34	30	24	37/63	96 (*R*)
35		48	33/67	97 (*R*)
36		72	25/75	97 (*R*)

Reaction conditions: (*RS*)-alcohol **2** (0.75 mmol), cells harvested from culture medium (100 mL) and ressuspended in phosphate buffer solution (0.1 mol/L, pH 7.0, 10 mL), 5–30 °C, 160 rpm, 24–72 h; Temperature for bacterial growth = 15 °C;

a Temperature for oxidation reaction;

b Determined by GC using a chiral column Chiral-Dex-CB.
